# Visual Imprinting in Birds: Behavior, Models, and Neural Mechanisms

**DOI:** 10.3389/fphys.2019.00658

**Published:** 2019-05-29

**Authors:** Brian J. McCabe

**Affiliations:** Sub-Department of Animal Behaviour, Department of Zoology, University of Cambridge, Cambridge, United Kingdom

**Keywords:** learning, memory, domestic chick, recognition, neural networks, perceptual learning

## Abstract

Filial imprinting is a process, readily observed in precocial birds, whereby a social attachment is established between a young animal and an object that is typically (although not necessarily) a parent. During a perinatal sensitive period, the young animal learns characteristics of the object (the imprinting stimulus) simply by being exposed to it and will subsequently recognize and selectively approach this stimulus. Imprinting can thus establish a filial bond with an individual adult: a form of social cohesion that may be crucial for survival. Behavioral predispositions can act together with the learning process of imprinting in the formation, maintenance, and modification of the filial bond. Memory of the imprinting stimulus, as well as being necessary for social recognition, is also used adaptively in perceptual classification of sensory signals. Abstract features of an imprinting stimulus, such as similarity or difference between stimulus components, can also be recognized. Studies of domestic chicks have elucidated the neural basis of much of the above behavior. This article discusses (1) principal behavioral characteristics of filial imprinting and related predispositions, (2) theoretical models that have been developed to account for this behavior, and (3) physiological results elucidating the underlying neural mechanisms. Interactions between these different levels of analysis have resulted in advancement of all of them. Taken together, the different approaches have helped define strategies for investigating mechanisms of learning, memory, and perception.

## Introduction

Filial imprinting has been recognized since antiquity and its behavioral characteristics reviewed extensively (Heinroth, 1911; Lorenz, 1935, 1937; Bateson, 1966; Sluckin, 1972; Hess, 1973; Bolhuis, 1991). It is readily observed in the young of many precocial species (i.e., where neonates are relatively mature and capable of locomotion in the immediate postnatal period) and most of the available information comes from newly hatched galliform birds such as chickens, ducks, and quail. Filial imprinting involves the young animal following a conspicuous stimulus, learning the stimulus’ characteristics, and consequently restricting its social preferences toward that stimulus. The consequences of filial imprinting can last well into later life and the phenomenon is generally adaptive, biasing the young animal’s behavior toward the protection of parents or other conspecifics. Sexual imprinting, whereby an animal’s sexual preferences are influenced by its previous experience, typically occurs around the time the animal assumes its adult appearance but may be influenced by experience in infancy. Thus, filial and sexual imprinting, though demonstrably distinct in terms of behavior, are interrelated (cf. Bolhuis et al., 1989).

Although imprinting occurs in a variety of sensory modalities, this article is mainly concerned with visual filial imprinting, which will be referred to simply as “imprinting” unless the term needs to be qualified to avoid ambiguity. Three interlinked perspectives will be considered. First, behavioral observations and experiments; second, theoretical models that have arisen from the behavioral work; and third, physiological experiments stimulated by the other two lines of research, which in principle permit theoretical predictions to be tested. The review discusses the contribution of imprinting to social cohesion in precocial avian species. It then describes theoretical models designed to account for key behavioral characteristics of imprinting. Finally, physiological data pertaining to mechanisms underlying the behavior are discussed. Such data may be used to test the models which, if validated, have the potential to be an explanatory link between the behavior and its underlying neural mechanisms.

## The Sensitive Period for Imprinting

Imprinting typically occurs within a perinatal sensitive period, which typically lasts for several days but which is very variable in duration (Bateson, 1966; Sluckin, 1972; Bolhuis, 1991). There is evidence that the beginning of the sensitive period depends, at least in part, on factors that are independent of sensory input (see also Section “The Sensitive Period for Imprinting” below). For example, sensitivity of ducklings to an imprinting stimulus was found by Gottlieb (1961) to be strongly associated with time since the start of embryonic development. It is also possible that time after hatching, and thus possibly the experience of hatching, contributes to the onset of the sensitive period (see also Williams, 1972; Hess, 1973; Landsberg, 1976). The degree of control exerted by developmental processes over the end of the sensitive period is less clear, but there is abundant evidence that imprinting itself can terminate the sensitive period. This is not to say that there is not an ontological termination of the sensitive period, since the end of the sensitive period is revealed behaviorally: the animal selectively approaches familiar objects and avoids novel objects as result of the social preferences acquired through imprinting. Such behavior does not necessarily reflect the ability to learn about an imprinting stimulus. The neural plasticity that is necessary for imprinting may, therefore, outlast the sensitive period (cf. Bateson, 1979), and there is evidence that this is indeed the case. For example, chicks that are already imprinted to an object and which avoid a second object, indicating behaviorally the end of the sensitive period, can eventually be imprinted to that second object (Salzen and Meyer, 1968; Cherfas and Scott, 1981; Bolhuis and Bateson, 1990).

### Predispositions

It would be surprising if all novel objects encountered by a naïve young animal were equally attractive and there is much evidence that different objects elicit different types and degrees of behavior in dark-reared chicks. For example, visually naïve chicks differentially approach objects of different colors (Bateson and Jaeckel, 1976). On finding a region in the chick forebrain (the IMM) that is critical for imprinting (see below), Horn and McCabe (1984) surveyed the results of several experiments in which this region had been ablated, and found that lesions to the IMM were very effective in eliminating preferences acquired through imprinting when the training stimulus was an artificial object such as a colored box or cylinder. However, the lesions were only partially effective when the training stimulus was a naturalistic object (the stuffed skin of an adult jungle fowl, resembling the presumed ancestral form). Subsequent experiments led to the conclusion that certain features of an imprinting stimulus elicit approach activity that does not depend on prior exposure to that particular stimulus. There is thus a predisposition to approach an object bearing these features. Dismantling the jungle fowl model and presenting its component parts in various positions and orientations implicated features in the head and/or neck as the critical targets of the predisposition (Johnson and Horn, 1988). Such features were later identified more precisely as the naturally occurring configuration of the eyes and mouth (Rosa-Salva et al., 2009). The predisposition is evidently triggered by mild interventions such as handling or exposure to light, as well as exposure to an imprinting stimulus (Johnson et al., 1985). The object of the predisposition is not restricted to conspecifics or congeners, since visually naïve chicks with the predisposition will preferentially approach a stuffed duck or polecat (Johnson and Horn, 1988). The predisposition evidently biases chicks’ approach behavior toward certain types of naturalistic stimulus, whereupon imprinting is available to establish a filial bond that is specific to the stimulus through learning. The relatively wide range of objects which the chick is predisposed to approach could be adaptive if the predisposition is activated only in a mildly stressful situation, such as social isolation, in an environment where there are more conspecifics than predators. The predisposition might then increase the probability of imprinting to a protective adult, albeit one that is not necessarily a close relative. Further predispositions have been described in newly hatched chicks, which suggest predilections for predictors of animacy, such as biological motion (Vallortigara et al., 2005), self-propulsion (Mascalzoni et al., 2010), the ability spontaneously to accelerate or decelerate (Rosa-Salva et al., 2016), alignment of an object’s major axis with its direction of motion (Rosa-Salva et al., 2018), and rotation (Rosa-Salva et al., 2018). Such stimuli tend to capture the animal’s attention and elicit approach behavior. The extent to which they lead to filial attachments by means of imprinting as opposed to predatory behavior, for example, remains to be determined.

Johnson and Bolhuis (1991) classified predispositions into general and specific, according to whether they are triggered by simple properties of a stimulus such as color (cf. Bateson and Jaeckel, 1976), or more complex combinations of features such as components of a face or biological motion as described above. Physiological experiments (see below) are helping to characterize these predispositions in terms of neural mechanisms. There is a striking similarity between chicks’ predisposition to approach faces and a predilection for face-like patterns in human neonates (see Di Giorgio et al., 2017 for review).

### Transient Preference for Novelty During Imprinting

A domestic chick’s preference for an imprinting stimulus during exposure to the stimulus (referred to as “training” in an experimental context) typically increases with duration of exposure (Bateson and Jaeckel, 1974; Zajonc et al., 1975). The temporal pattern of this increase in preference need not be linear. Under controlled training conditions and low variation in chicks’ rate of approach, a transient preference for novelty was found to emerge before a strong preference for the training stimulus became established (Bateson and Jaeckel, 1976). These authors suggested that the transient reversal of preference results from a tendency to prefer slight novelty once they have become familiar with a training stimulus after a brief encounter (about 15 min in the experiments in question). This might be adaptive if the chicks were thereby prompted to explore slightly novel stimuli, such as different views of an imprinting object, while many features of the stimuli, such as color, appear to remain relatively constant. Under natural circumstances, when the imprinting stimulus is a mother hen, a chick might first become attracted to one view of the hen and later prefer a different view. A progressive series of such events could cause the chick to become familiar with many views of the hen, aiding recognition of the hen from several viewing angles. This idea was tested by Jackson and Bateson (1974), who trained chicks with either a red or a yellow stimulus and then allowed the chicks to choose a stimulus of either color by pressing a pedal. Consistent with the prediction, after 15 min exposure, the chicks actively worked to obtain exposure to the novel color. After 30 min exposure, a similar but weaker trend was observed and after 60 min, chicks chose the familiar color. In natural conditions, this type of behavior might be expected to bias chicks’ behavior toward a slightly novel view of a mother hen, thus obtaining information about different views of her, facilitating recognition of the mother from different viewpoints in different viewing conditions. This hypothesis is supported by the results of Honey and Bateson (1996), which imprinted chicks on the side and back views of a hen in rapid temporal succession and found that the chicks took longer to learn the difference between these two views than chicks trained on the two views separated by much longer intervals. The results thus suggest that a stronger perceptual link is formed between two stimuli, the more rapidly one stimulus is presented after the other.

### Classification Together of Temporally Juxtaposed Stimuli

It is noteworthy that a theoretical model predicting a temporary preference for slight novelty (Bateson, 1973, 1974; see below) implies a time-dependent perceptual modification, which determines whether a chick classifies any particular stimulus together with the familiar training stimulus. The possibility of classification together was also raised by behavioral experiments in which two visual imprinting stimuli were shown to chicks according to different schedules. Chantrey (1974) trained chicks by exposing them to two visual imprinting stimuli, presented alternately. He found that rapid alternation with a short inter-onset time (e.g., 7 s exposure to stimulus A, 8 s exposure to no stimulus, 7 s exposure to stimulus B, etc.) had a different effect from a longer inter-onset time (e.g., 30 min exposure interspersed with 30 min exposure to no stimulus). Total amount of exposure to each stimulus was kept constant. The difference lay in the ease with which the chicks could subsequently learn to distinguish stimulus A from stimulus B in an operant training procedure when one of these stimuli was associated with food reward. With shorter inter-onset times, the discrimination was learned more slowly. This result prompted the hypothesis that rapid alternate exposure to the two stimuli caused them to be classified together. In support of this interpretation, Chantrey (1976) also found that rapidly alternating exposure of chicks to two different colored stimuli led to similar behavior toward the two stimuli, whereas a longer inter-onset time caused the two stimuli to elicit different behaviors; see also Honey and Bateson (1996), discussed above. If the interpretation of classification together is correct, rapidly alternating views of different parts of a mother hen during imprinting might cause a chick to classify these different views together, so that the hen was approached subsequently irrespective of which view was momentarily presented to the chick. It is noteworthy that such a process could in principle be facilitated by preference for slight novelty during imprinting (see above).

### Imprinting to Several Objects

During infancy, a young animal typically encounters a wide range of stimuli, raising the question of how stimuli that are appropriate for filial bonding may be distinguished from those that are not, including those which may actually be harmful. Available possibilities include predispositions to cleave to appropriate objects (e.g., parents), familiarity as a result of prolonged exposure to these objects and reinforcement of behavior that brings the infant into close contact with them by such factors as warmth and somatosensory stimulation. Given that a parent is often the first object to be seen after hatching, one might also suppose that order of exposure is important in molding a young animal’s subsequent filial behavior. Salzen and Meyer (1968) imprinted chicks, first with either a green or a blue ball and later with the alternative object, i.e., either a green ball followed by a blue ball or vice versa. The chicks were given repeated test choices between the two objects. A strong preference was readily acquired for the first object encountered and the preference was later reversed after prolonged exposure to the alternative object. Imprinting can therefore be reversed by sequential exposure to two stimuli. The question was reexamined by Cherfas and Scott (1981), who also found a reversal of preference, but additionally found reemergence of a preference for the first stimulus if chicks were isolated for 3 days after exposure to the second stimulus. It was unclear whether the reemergence of the original preference was due to forgetting of both stimuli and a predisposed bias toward one of them. Bolhuis and Bateson (1990) addressed this issue using two disparate training stimuli and found that a preference for the stimulus of first exposure eventually recurred irrespective of the order in which the two stimuli were presented. There is therefore a primacy effect: the first filial attachment can be over-ridden but there is a tendency to revert to this original attachment with time. Notwithstanding this reversion, it is still possible after secondary imprinting to imprint to a third stimulus (Devos and Vankampen, 1993), consistent with the idea that imprinting can update representations in the course of establishing a filial bond.

### Relational Concept Learning

Exposure of young ducklings to a moving visual stimulus for about 30 min results in a predicable filial attachment to the stimulus (Martinho and Kacelnik, 2016). The ducklings clearly recognized the stimulus after exposure, but the question arises as to what type of information contributes to recognition. One possibility is simple morphological template matching between the training stimulus and what is learned during training. There is, however, the alternative possibility that abstract features of the stimulus are stored and used for recognition. Such a process might offer the advantage of economy of coding: rather than a point-by-point representation of the stimulus, more general features such as size, color, and symmetry might efficiently be encoded as critical features of the stimulus. Martinho and Kacelnik (2016) trained ducklings with an imprinting stimulus comprising two halves, possessing either the same or different forms, or the same or different colors. The ducklings were then tested to find out whether they had acquired a preference for “sameness” or “difference” by giving a duckling a choice between two novel stimuli, one comprising two parts that were the same and the other in which the two parts were different. The results indeed indicate that the relational properties of the stimuli, i.e., “sameness” or “difference,” can be encoded and used as a basis for discrimination.

## Models of Imprinting

### Model 1

**Figure 1:**
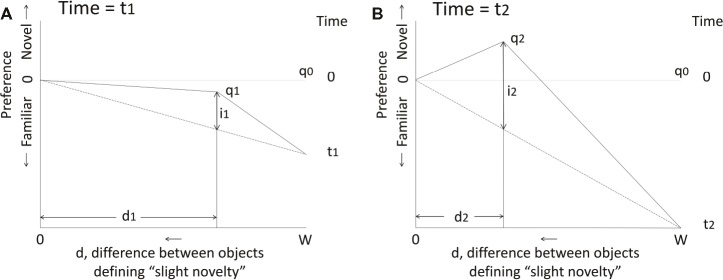
Predictions of the simplest form of the model of Bateson (1973, 1974). On both graphs, the vertical axis represents the chick’s preference during imprinting to a training object and the horizontal axis represents the difference *d* between the training object and any other object that is perceived as “slightly novel”; “slight novelty” corresponds to a level of novelty that is maximally attractive. *d* ranges between zero and *W*, the width of the stimulus continuum. Before imprinting starts, the model has no preference for any object: all are equally novel, equally attractive and *d* = *W*. After imprinting has started, preference for the training object increases linearly with time (*t*). *d* decreases linearly with time, as the chick becomes more familiar with the training object and is able to discriminate it better from a novel object. Point *q* represents the chick’s state as *d* changes with time. The attractiveness of “slight novelty” is given by *i*, which also increases linearly with time until discrimination is so good that *d* goes to zero and “slight novelty” ceases to exist. Thereafter, preference for familiar continues to increase unopposed to some limiting value. **(A)** State *q*_1_ at time *t*_1_. A preference for the now familiar training object has been acquired and the influence of “slight novelty,” given by *i*_1_, is not yet strong enough to oppose the acquired preference for familiar; there is therefore a net preference for the training object relative to all others, including the maximally attractive “slightly novel” object. The position *q*_1_ is defined by *d*_1_ and *i*_1_. **(B)** State *q*_2_ at time *t*_2_. Preference for the familiar training object over any other object has increased further, but the preference for “slight novelty” has increased sufficiently to give a net preference for “slight novelty” over the training object. At some further time, *d* will decrease to zero and preference for the familiar training object is thereafter unopposed. See Bateson (1973, 1974) for further details, including adjustments to account for real-world complications such as biases in the preferences of naïve chicks.

Transient reversal of preference during imprinting was explored in a theoretical model by Bateson (1973, 1974). The model is highly simplified but shows how two processes that simultaneously progress with time during training can act together to produce a temporary preference for slight novelty. In its simplest form, the model assumes that a naïve chick is equally responsive to all objects, even two stimuli that are so disparate as to be at opposite ends of the stimulus continuum. Exposure to one such object (the training object) increases a chick’s responsiveness to that object as training progresses. Also increasing with exposure time is the chick’s responsiveness to a stimulus that is perceived as slightly different from the training stimulus and which, as such, is maximally attractive. Finally, the difference between two stimuli that is required for the stimuli to be perceived as different decreases with exposure time (Figure 1). With appropriate choice of parameters, the model predicts: (1) a transient preference for slight novelty as training progresses. It also predicts (2) that when an alternative stimulus is used in a choice test with the training stimulus, the more similar the stimuli in the test, the later the transient preference for novelty will occur. The first prediction was consistent with the results of Bateson and Jaeckel (1976) in a direct test of the prediction; the second prediction has yet to be rigorously tested owing to the difficulty of obtaining stimuli with suitable levels of disparity from one another.

The model thus accounts for the observation that a training stimulus becomes more attractive as it becomes familiar, but also for the finding that a slightly different stimulus can transiently become more attractive still. A further feature of the model is that the threshold for perception of slight novelty decreases as training progresses; this seems plausible given that more time spent observing the training stimulus gives more opportunity to learn about it. By implication, two stimuli differing by less than this threshold would elicit the same level of response and effectively be classified together by the chick.

### Model 2

**Figure 2:**
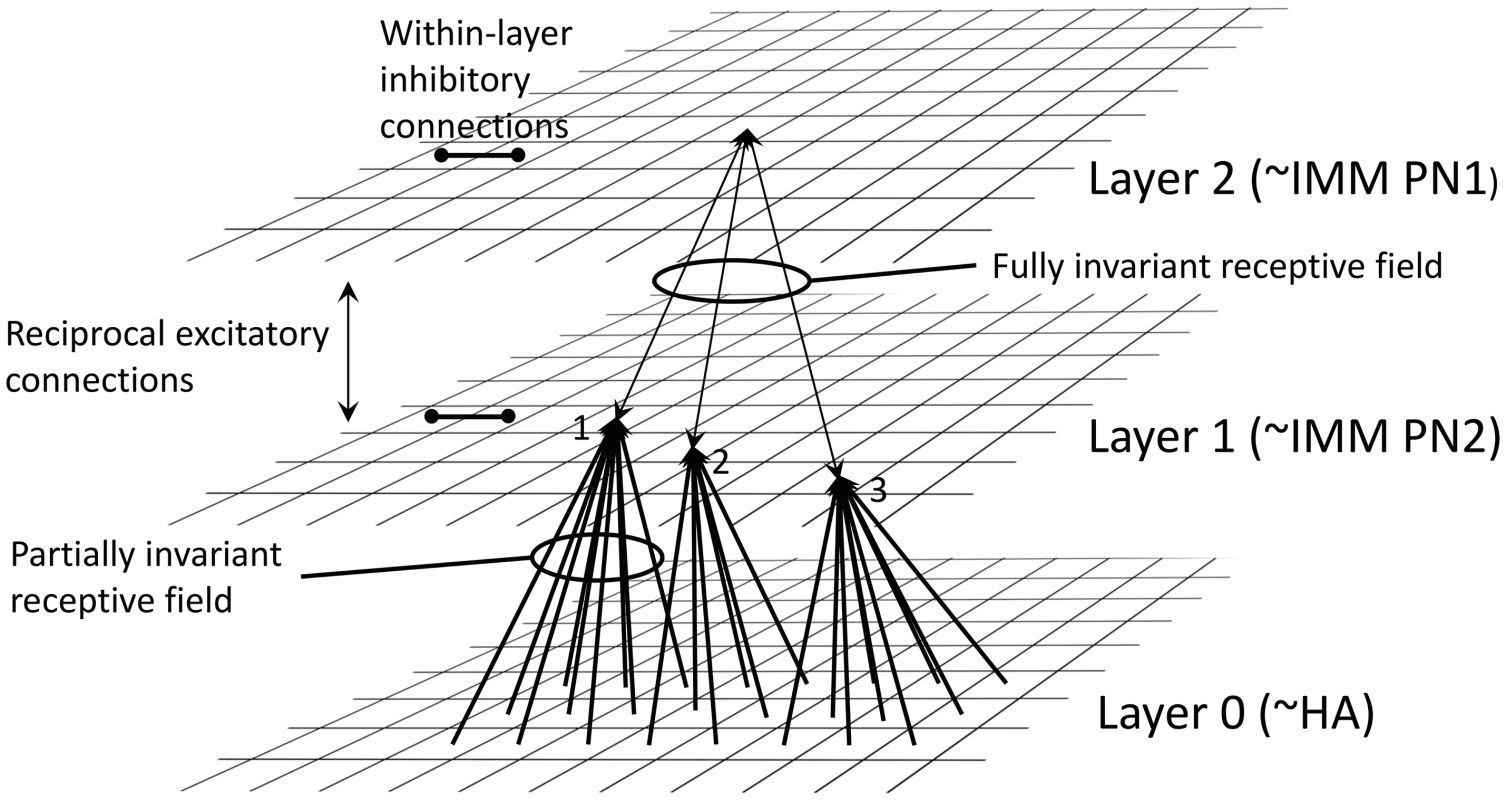
Architecture of the neural net model of O’Reilly and Johnson (1994), redrawn from their paper. Layer 0 is suggested to correspond to the visual wulst, including the hyperpallium apicale (HA). Connections from units in layer 0 converge *via* Hebbian synapses onto units in Layer 1, suggested to correspond to part of the IMM. There is further projection *via* Hebbian synapses to Layer 2, also in the IMM. Two types of projection neuron (PN) are proposed: some axons from PN1 neurons are suggested to project out of the IMM, and PN2 neurons are suggested to be intrinsic to the IMM. Reciprocal excitatory connections combined with hysteresis render the receptive fields in Layer 2 fully position-invariant. Mutual inhibition between units within Layers 1 and 2 is also a necessary feature of the model. See O’Reilly and Johnson (1994) for details.

The model of O’Reilly and Johnson (1994) is a neural network comprising an input layer (layer 0, corresponding to the hyperpallial visual projection area of the forebrain, receiving visual input from the lateral geniculate nucleus of the thalamus) and two further layers (1 and 2), corresponding to different components of the IMM (cf. Section “Transient preference for novelty during imprinting”). Layer 0, which contains units with properties of simple and complex visual cortical cells as found in mammals (see e.g., Douglas and Martin, 2004), sends converging excitatory inputs to layer 1, which in turn sends converging excitatory inputs to layer 2 (Figure 2). The effect of this cascading configuration is to preserve the features of the visual imprinting stimulus but as a representation that is invariant with respect to retinal position. The inputs to layers 1 and 2 bear modifiable synapses that obey a Hebbian rule (Hebb, 1949), namely that conjoint pre- and post-synaptic activity strengthens the synapse such that coincident inputs on a post-synaptic cell are strengthened. There is reciprocal excitatory feedback from layer 2 to layer 1 and lateral inhibition between neighboring units in layer 2. Neurons in the model exhibit hysteresis, namely persistence of activity after activation. The properties of Hebbian plasticity and hysteresis, with suitable parameters, convey biologically realistic properties on the model and make the following predictions:

1. There is a translation-invariate representation of the training stimulus within the IMM.
2. Selective modification of connections leading to discrimination between familiar and unfamiliar stimuli.
3. A sensitive period for learning that terminates once learning has progressed to a certain level.
4. Limited reversibility of imprinting on exposure to a second training stimulus.
5. Residual recognition of the first training stimulus after training with a second training stimulus.
6. Generalization between a training stimulus and other, similar stimuli.
7. The inability to discriminate between two different training stimuli if they are present in close temporal contiguity, termed “temporal blending” by O’Reilly and Johnson (1994).
8. For given high level of temporal contiguity with which two training stimuli are presented, the more similar the stimuli, the lower the level of temporal blending. The opposite is the case if there is more delay between alternate stimulus presentations.

Properties (1)–(7) have been demonstrated in behavioral experiments (cf. Bateson, 1966; Sluckin, 1972; Horn, 1985; Bolhuis, 1991). Property (8) may seem counter-intuitive: one might expect similar stimuli to be consistently highly prone to temporal blending when presented serially with a very short delay. Indeed, Chantrey (1974) and Honey and Bateson (1996) found evidence for temporal blending in chicks trained with alternate serial presentation with two imprinting stimuli. However, the effect was not found with a different pair of training stimuli and different experimental conditions (Stewart et al., 1977), possibly due to conflicts implied in the simulation.

### Model 3

**Figure 3:**
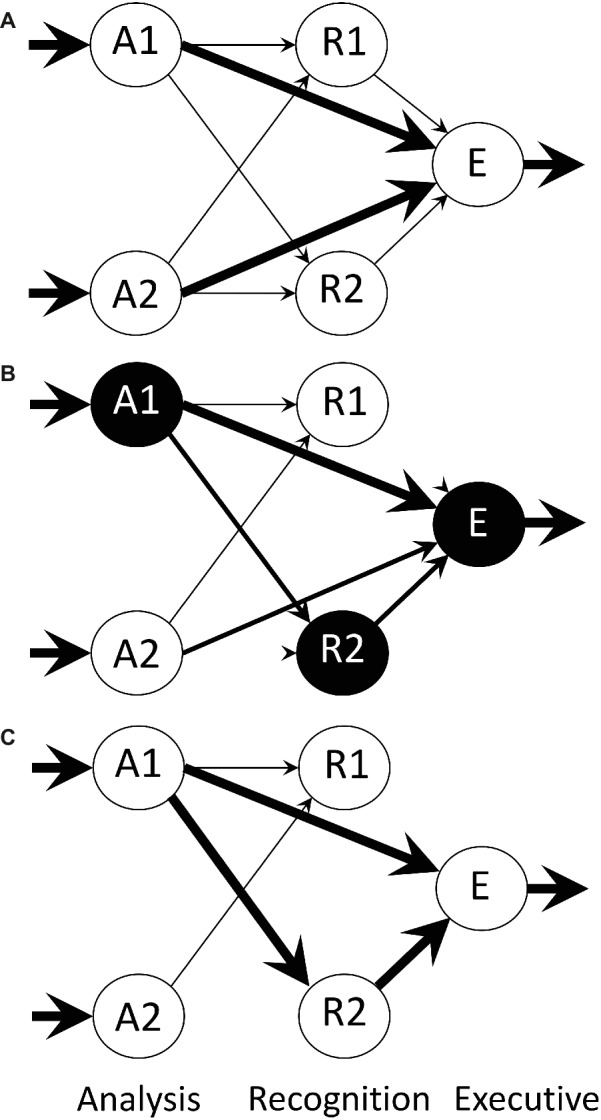
Architecture of the neural net model of Bateson and Horn (1994), from an illustration by Bateson (2015). The model comprises three layers: A (analysis), R (recognition) and E (executive). The A and R layers contain a number of modules, only two of which are shown here. Each A module relays processed sensory input bearing information about the training object, such as size, shape, color, etc., the arrows indicate flow of information, and the thicknesses of the arrows represent connection strength of the Hebbian synapses at the head of each arrow. Within each of the A and R layers, there is reciprocal inhibition *via* non-modifiable connections between modules (not shown). There are also direct Hebbian links from analysis to executive, by-passing the R layer, permitting a predisposition to be expressed and allowing simple conditioning outside the R layer. Modules are spontaneously and variably active and mutual inhibition within a layer permits the representation of an imprinting stimulus to be encoded by strengthening the more active pathways. The activity of these pathways weakens alternative inactive pathways. **(A)** Initial state of the network before training. **(B)** Network during training with an imprinting stimulus that activates analysis module A1. Recognition module R2 happens to be highly active when input from A1 first arrives and consequently ‘captures’ that input while suppressing other recognition modules. **(C)** Network after training is complete.

Bateson and Horn (1994) describe a neural network implementation of a model of imprinting developed previously (Bateson, 1981, 1991; Horn, 1985). The model possesses three layers: analysis, recognition, and executive, each layer containing a set of modules acting in parallel (Figure 3). Analysis modules respond selectively to particular aspects of a stimulus such as size, shape, and color and evidently have acquired the position-invariance such as can be achieved by the process incorporated into the model of O’Reilly and Johnson (1994). In the naïve state, each analysis module is connected to each module in the recognition layer by links that can be modified in an activity-dependent manner. Each recognition module is in turn linked to an executive module by modifiable convergent connections. An executive module controls filial approach behavior toward the stimulus, as either approach or withdrawal behavior. As well as information about an imprinting stimulus flowing from analysis to recognition to executive through modifiable links, there is a direct pathway from analysis to executive, by-passing recognition and also containing modifiable links.

The rule by which a modifiable link between modules can be strengthened is Hebbian (Hebb, 1949), in that strengthening occurs if both input and recipient components of the link are simultaneously active. A link is weakened if the recipient component is active and the input is inactive, a principle arising from studies of activity-dependent plasticity in developing sensory systems (Stent, 1973). When the recipient component is inactive, the strength of the link does not change, irrespective of the state of the input. Input to a recognition module from an analysis module results in: (1) activation of an intrinsic excitatory unit, which in turn activates an output unit projecting to an executive module, all *via* modifiable links; (2) activation of a unit that inhibits the output unit (Figure 3), non-modifiably. Modules within a layer inhibit each other reciprocally via non-modifiable links. When the animal is in the naïve state, the activity of each recognition module fluctuates spontaneously.

With suitable choice of parameters, the model reproduces a considerable number of behavioral results, in particular:

1. acquisition of preference for an imprinting stimulus, including where stimuli differ in their attractiveness before training;
2. acquisition of a preference for inconspicuous details of a stimulus when paired with conspicuous stimulus details, as happens when imprinting leads to recognition of individual animals (Johnson and Horn, 1986b);
3. stimulus generalization after imprinting (Jackson, 1974; Bolhuis and Horn, 1992);
4. a sensitive period for imprinting that is closed by imprinting itself (cf. Bolhuis, 1991);
5. classification of a stimulus on the basis of only a subset of its features, and when the contents of the subset changes – a so-called “polymorphous category” (cf. Von Fersen and Lea, 1990);
6. classification together of different stimuli, either when the stimuli are presented simultaneously (Bateson and Chantrey, 1972; Chantrey, 1972) or in a rapid temporal sequence (Chantrey, 1974);
7. the updating of a stimulus representation when the stimulus gradually changes and certain of its features disappear with time (Bateson, 1979);
8. the persistence of the ability to learn a task requiring discrimination between two visual stimuli after ablation of a brain region (the IMM) that is necessary for imprinting (Johnson and Horn, 1986a,b);
9. the formation of associative links between different stimuli by conditioning outwith the IMM (McCabe et al., 1982);
10. the expression of predispositions to approach certain classes of stimulus.

Estimation of the model’s parameter values has been attempted. This of course makes the assumption, yet to be comprehensively tested, that the model is physiologically valid. The modifiable links in the model can be strengthened by use or weakened by inhibition from another pathway. Strengthening was set in the model to four times as strong as weakening. Griffiths (1998) trained chicks for 120 min with stimulus A. A control group received no further training while an experimental group received a further 180 min of training with stimulus B. Chicks were then tested by being given a choice between either A and B or A and C, which had not previously been seen. Using the control chicks as a standard for comparison, further training with B reduced preference for A against C, assumed to be due to weakening of links encoding A by exposure to C. Further training with B caused a greater reduction of preference for the red triangle against B, assumed to be due to weakening of links encoding A plus strengthening links for B. Notwithstanding all the assumptions, the strengthening to weakening ratio thus determined was estimated as 4.3:1, corresponding closely the value of 4 assumed in the model.

## Physiological Mechanisms

### Memory for the Imprinting Stimulus

**Figure 4:**
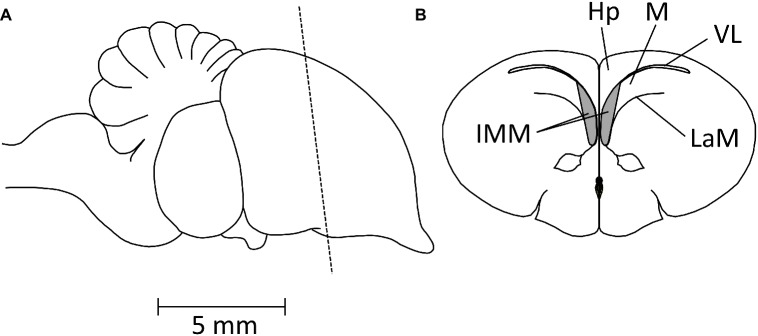
Diagrams showing the position of the IMM. **(A)** Side view of chick brain, anterior pole to the right. The broken line indicates the plane of the coronal section shown in **(B)**. IMM, intermediate and medial mesopallium; Hp, hippocampus; M, mesopallium; VL, lateral ventricle; LaM, medullary lamina.

Bateson, Horn, and Rose conducted a series of experiments on domestic chicks to determine whether neural changes could be detected that were specifically related to the learning that occurred in the course of imprinting. Evidence for such a change was found in the forebrain (reviewed in Horn et al., 1973) and further localized by Horn et al. (1979) to a restricted part of the forebrain roof, the intermediate and medial mesopallium (IMM), known as the IMHV before revision of avian brain nomenclature by Reiner et al. (2004). The position of the IMM is shown in Figure 4.

Horn (1985) gives a detailed account of the evidence that the IMM is a site of memory for features of a visual imprinting stimulus. This evidence includes lesion studies indicating that the IMM is necessary for both acquisition (McCabe et al., 1981) and retention (McCabe et al., 1982) of a preference through imprinting, and that an increase in the area of apposition of spine synapses was observed after imprinting training (Bradley et al., 1981; Horn et al., 1985); this morphological change was lateralized to the left side of the IMM. The preferential involvement of the left side of the IMM in learning-related changes after imprinting has been a common occurrence over many studies and is consistent with hemispheric asymmetries found in lesion studies of the IMM (Horn, 1985; McCabe, 1991; Solomonia and McCabe, 2015).

Since spine synapses are often excitatory (see e.g., Nafstad, 1967; Errington et al., 1987), receptors for the excitatory neurotransmitter L-glutamate were studied in the IMM, and a localized learning-related increase in numbers of N-methyl-D-aspartate (NMDA) receptors in the left IMM was found after imprinting training (McCabe and Horn, 1988, 1991); see McCabe (2013) and Margvelani et al. (2018) for a discussion of how one might infer that a neural change observed after training is associated with learning and/or memory. NMDA receptors in the IMM are evidently necessary for learning, since local injection of the competitive NMDA receptor blocker D-AP5 at an estimated concentration specific for NMDA receptor blockade prevented imprinting without detectable effect on visuomotor capabilities (McCabe et al., 1992). Calcium-dependent, potassium-stimulated release of L-glutamate from the IMM also rose after imprinting training, although not in a manner specifically related to learning (Meredith et al., 2004). Learning-related phosphorylation of a glutamate receptor, namely the GluA1 subunit of the ionotropic alpha-amino-3-hydroxy-5-methyl-4-isoxazolepropionic acid (AMPA) receptor has, however, been detected (Solomonia et al., 2013).

Immunocytochemical labeling of the activity marker c-fos protein identified a population of neurons in the IMM that were specifically activated when a chick learned about an imprinting stimulus (McCabe and Horn, 1994). Almost all these neurons were immunopositive for taurine (Potter et al., 1998), the inhibitory neurotransmitter gamma-aminobutyric acid (GABA) and the calcium-binding protein parvalbumin, but not for the calcium-binding protein calbindin (Ambalavanar et al., 1999). An sub-population of IMM neurons associated with imprinting memory has thus been identified. Interestingly, imprinting also gives rise to presumed synaptic release of both GABA and taurine from the IMM (McCabe et al., 2001; Meredith et al., 2004).

Learning-related changes in presumed synaptic physiology in the IMM following imprinting were classified by Solomonia and McCabe (2015) into early, intermediate and late according to whether they occurred up to approximately 7 h, 7–15 h or > 15 h, respectively, after training with an imprinting stimulus. Early changes include enhanced calcium-dependent release of GABA and taurine (McCabe et al., 2001), possibly under the control of phosphorylated myristoylated alanine-rich C-kinase substrate (MARCKS protein) (Sheu et al., 1993; Van der Zee et al., 1995; Solomonia et al., 2003, 2008), and released from inhibitory neurons that are immunopositive for parvalbumin and protein kinase C-gamma but not calbindin (Ambalavanar et al., 1993, 1999). In this early period, there is also up-regulation of autophosphorylated calcium/calmodulin-dependent protein kinase II (CaMKII) (Solomonia et al., 2005), responsible for increased phosphorylation of Serine 831 in the GluA1 subunit of the AMPA glutamate receptor. Calcium-dependent release of L-glutamate in the IMM is also increased in this early period, but not quantitatively correlated with the strength of learning. Therefore, any learning-related modulation of glutamatergic activity is likely to be by modification of AMPA receptors. Calcium-dependent release of GABA and taurine continue to be enhanced in the intermediate period (7–15 h after the end of training), but accompanied by a non-specific down-regulation of the gamma-4 subtype of GABA receptor (Harvey et al., 1998). An up-regulation of the NMDA subtype of glutamate receptor that is correlated with preference score is also observed, restricted to the left IMM (McCabe and Horn, 1988). In the late period (>15 h after the end of training), there is evidence of trophic changes and synaptic stabilization approximately in proportion to the amount learned by the chicks, namely up-regulation of neural cell adhesion molecules (Solomonia et al., 1998), clathrin (Solomonia et al., 1997) and amyloid precursor protein (Solomonia et al., 2003) and cognin/brain spectrin (Meparishvili et al., 2015). Other learning-related changes at this time are suggestive of membrane and cytoskeletal stabilization, implicating alpha-fodrin (Solomonia et al., 2011) and MARCKS (Solomonia et al., 2003, 2008).

Learning-related changes in the IMM have been found in mitochondrial proteins: subunits I and II of cytochrome c oxidase, a critical enzyme in oxidative metabolism, were found to be up-regulated in the left IMM (Solomonia et al., 2011). These subunits are encoded by mitochondrial DNA (Khalimonchuk and Rodel, 2005). Further study of proteins in the mitochondrial/membrane fraction from the IMM revealed learning-related changes, restricted to the left IMM, in (1) membrane cognin; (2) a protein resembling the P32 subunit of splicing factor SF2; (3) voltage-dependent anionic channel-1; (4) dynamin-1; and (5) heterogeneous nuclear ribonucleoprotein A2/B1. There were also, in the left IMM, learning-related changes in transcription factors involved in mitochondrial biosynthesis without significant change in DNA copy number (Meparishvili et al., 2015). These changes are accompanied by increased rates of mitochondrial fission and fusion, but these processes were balanced, indicating that overall numbers of mitochondria in the IMM are stable 24 h after imprinting training. See Solomonia and McCabe (2015) for a summary of learning-related biochemical changes in the IMM following imprinting.

Regulation of protein synthesis was investigated by enquiring whether there were changes in levels of micro RNAs (miRNAs), which inhibit protein synthesis by pairing with bases in the 3′-untranslated regions of mRNA and either blocking translation into protein or destroying the RNA. A preliminary screen using the left IMMs from a small number of strongly or weakly imprinted chicks implicated a particular miRNA in imprinting on the basis of statistical significance and expression level. Levels of this miRNA (gga-miR-130b-3p) in the left IMM were negatively correlated with preference score and a range of criteria implicated the miRNA in a predisposition to learn (Margvelani et al., 2018; see Section “A Predisposition to Learn”). One of the protein products of this molecule is cytoplasmic polyadenylation binding protein 3 (CPEB-3), and this protein was significantly up-regulated in a learning-related manner in the left IMM as a result of imprinting training (Margvelani et al., 2018). This is thus an example of a miRNA in the IMM that is unaffected by imprinting but which predisposes a chick to learn, whereupon the protein whose synthesis is controlled by the same miRNA is up-regulated as a result of training and is intimately involved in the memory 24 h after training.

As well as the evidence for learning-related functional synaptic modification in the IMM, particularly on the left side of this structure, single unit recording in freely moving chicks has shown that neuronal responsiveness in the IMM to a visual imprinting stimulus increases as a result of imprinting training (Brown and Horn, 1994; Nicol et al., 1995; Horn et al., 2001; Nicol and Horn, 2009). Training was conducted in the presence of a hen’s maternal call in order to render imprinting to the visual stimulus more effective (Bolhuis and Honey, 1994). Testing, however, was purely visual. As might be expected from morphological, pharmacological, and biochemical findings (Horn, 1985; McCabe, 1991, 2013), different effects of training were found in the left and right sides of the IMM (Nicol et al., 1995). When the visual and auditory components of the bisensory training stimulus were presented separately after training, it was found that neuronal responsiveness to the visual component had increased, whereas responsiveness to the maternal call was reduced (Nicol and Horn, 2009). The enhanced neuronal responsiveness to the visual component was confirmed by Town (Town, 2011b), who found further that this enhancement was reduced if chicks, having been trained originally in isolation, were then reared with conspecific chicks for 9 h. The reduction was largely in the left IMM, and an increased response to a novel stimulus was observed in the right IMM. The social rearing also reduced chicks’ behavioral preferences for the original training stimulus, as might be expected from previous studies of secondary imprinting (Salzen and Meyer, 1968; Cherfas and Scott, 1981; Bolhuis and Bateson, 1990; Devos and Vankampen, 1993). Neuronal responsiveness in the IMM thus changes in parallel with behavioral preferences when a second imprinting stimulus is introduced.

The pathway whereby visual information reaches the IMM was investigated by Nakamori et al. (2010), who identified a polysynaptic thalamofugal visual pathway reaching the IMM *via* synaptic connections in the interstitial nucleus of the hyperpallium apicale, and in the rostral, densocellular and periventricular parts of the hyperpallium dorsale (HD). Imprinting was associated with NR2B-containing NMDA receptors that contribute plasticity in this circuit (see also Nakamori et al., 2015). Cholecystokinin has been implicated in the role of the visual wulst in imprinting (Maekawa et al., 2007) and there is selective activation of presumed GABA-ergic parvalbumin-containing cells in this pathway (Nakamori et al., 2017).

#### The Effect of Sleep on Imprinting Memory

Experiments on human subjects have established that sleep after certain types of learning can enhance subsequent recall of the learned items (cf. Vorster and Born, 2015), but the underlying neural mechanisms are imperfectly understood. It was suggested (Horn et al., 2001) that neuronal responses in the IMM to a familiar imprinting stimulus may become stabilized by sleep, but this was not established until Jackson et al. (2008) recorded single units from the IMM during and after imprinting and found that undisturbed sleep within a particular temporal window after imprinting training was necessary for both stability of neuronal responsiveness to the imprinting stimulus and retention of the imprinted preference measured behaviorally. It was already known that if chicks were allowed to rest in darkness over a 6-h period after imprinting training for 2 h, responsiveness to the imprinting stimulus was enhanced significantly 24 h after the start of training (Horn et al., 2001). However, if continuous sleep was prevented during this period by occasional gentle disturbance (slowly rotating the running wheel in which the chicks were held in darkness once every 30 min), neuronal responses were unstable – neurons that had once been responsive ceased to respond and previously unresponsive neurons started to respond. This instability persisted at 24 h and chicks showed no evidence of being imprinted at that time. However, if the same disturbance was delayed for 6 h, by the 24-h time point, the neuronal response had stabilized at a significantly higher level and the chicks showed a behavioral preference for the imprinting stimulus. There was increased activity in the lower theta range (5–6 Hz) of the electroencephalogram during the 6-h period after training had finished, during which sleep disruption was effective in disrupting both neuronal responsiveness and retention of the preference acquired through imprinting (Jackson et al., 2008). In view of the finding of Marshall et al. (2004) and Marshall et al. (2006) that transcranial electrical stimulation of the human brain at a frequency of 0.75 Hz (corresponding to the frequency of slow-wave sleep) enhanced declarative memory recall, Nicol and McCabe (2014) electrically stimulated the brains of chicks at 5 Hz [the frequency enhanced during the post-training sleep period in the experiments of Jackson et al. (2008)] or 0.75 Hz (the main frequency of slow-wave sleep) during the 6-h period after training when sleep was necessary for stabilization of IMM neuronal responsiveness and behavioral retention. Stimulation at both these frequencies protected against loss of the preference acquired during imprinting.

O’Reilly and Johnson (1994) found that the responsiveness of units in Layers 1 and 2 of their neural network model became unstable in the early stages of imprinting as the balance between strengthening and weakening of connections was becoming established. It is therefore noteworthy that instability in neuronal responsiveness in the IMM was observed shortly after imprinting by Horn et al. (2001) and that this instability was strongly reduced by sleep in a specific 6-h period after the end of training (Jackson et al., 2008).

The models of O’Reilly and Johnson and Bateson and Horn (1994) rely on Hebbian plasticity, and therefore it is appropriate to enquire where such plasticity has been described in the IMM. Associative long-term potentiation and depression are well-established forms of synaptic plasticity based on Hebbian mechanisms, which depends on activation of NMDA receptors (see e.g., Morris, 2003). Investigation of the IMM *in vitro* has revealed plasticity resembling glutamate receptor-mediated long-term potentiation (Bradley et al., 1991, 1993, reviewed in Matsushima and Aoki, 1995; Bradley et al., 1999) and susceptibility of imprinting to local pharmacological blockade of NMDA receptors in the IMM (Mccabe et al., 1992).

### The Sensitive Period for Imprinting

Mechanisms underlying the sensitive period have been studied by correlational measurements, determining which physiological changes parallel the sensitive period, in combination with physiological and pharmacological interventions to modify the sensitive period.

The start of the sensitive period for visual imprinting is clearly dependent on the ontogeny of the neural systems necessary for perception of an imprinting stimulus, together with motivational and motor systems required to express the imprinted behavior. This is not to say that experience does not influence the sensitive period, since sensory stimulation is necessary for complete development of sensory systems, which are themselves subject to sensitive periods (see e.g., Feldman, 2009). A detailed example of an effect of sensory stimulation on a sensitive period comes from experiments on auditory imprinting in ducklings, where post-hatch ability to acquire a preference for the conspecific adult maternal call requires previous experience of embryonic contact-calls in the egg (reviewed in Gottlieb, 1987). Moreover, the ability to imprint to a non-conspecific call (the maternal call of a chicken) was not detected in ducklings reared in isolation, but only occurred when ducklings experienced tactile contact during social rearing (Gottlieb, 1993). An influence of visual experience on subsequent visual imprinting was described by Bateson and Wainwright (1972), who found that exposure of chicks hatched and reared in darkness to white light for 30 min before exposure to imprinting stimuli increased the efficacy of visual imprinting. This effect was tentatively ascribed to activation of visual pathways by the white light.

Barbiturate anesthesia has been found to extend the sensitive period (Macdonald, 1968). An anesthetic dose of a ketamine/xylazine mixture administered on the day of hatching enabled chicks to become imprinted to a jungle fowl model on day 8 post-hatch, whereas this did not occur in saline-treated controls (Parsons and Rogers, 1997). This effect was later ascribed to the inhibitory action of ketamine on the NMDA subtype of glutamate receptor, since the effect was reproduced using the specific NMDA receptor antagonist MK-801 (Parsons and Rogers, 2000). It is worth noting that the sensitive period in these experiments was defined in terms of a preference for a model of a fowl, raising the possibility that specific patterns within this naturalistic stimulus may have been especially important in these experiments.

Thyroid hormone has been strongly implicated in control of the sensitive period for imprinting in chicks. It was reported by McNabb (2006) that thyroid hormone levels peak around the time of hatching in precocial birds. Also, the gene for Type 2 iodothyronine deiodinase (Dio2), the enzyme that catalyzes conversion of thyroxine (T4) to triiodothyronine (T3), is up-regulated when chicks become imprinted (Yamaguchi et al., 2008). Yamaguchi et al. (2012) also found that T3 levels in brain peaked around hatching and increased as a result of imprinting. Dio2 inhibitors administered either systemically or into the IMM, blocked visual imprinting and T3, and administered either intravenously or into the IMM could increase the efficacy of a visual imprinting stimulus and extend the sensitive period for several days. Moreover, both imprinting and exogenous T3 facilitated imprinting on a second stimulus (Yamaguchi et al., 2012), suggesting that T3 might contribute to neural mechanisms underlying updating of the representation of an imprinting stimulus. Nucleotide diphosphate kinase 2 has been implicated in the action of T3 by the demonstration that inhibition of this enzyme in the IMM blocks the action of T3 in extending the sensitive period (Yamaguchi et al., 2016). The intermediate hyperpallium apicale (IMHA) receives output from the IMM and has been implicated in imprinting by Aoki et al. (2015). The IMHA receives a projection from the IMM and is the site of an increase in the level of Wnt-2b, a glycoprotein that regulates neuronal growth, when imprinting occurs (Yamaguchi et al., 2018). Blockade of Wnt-2b action in the IMHA prevents expansion of the sensitive period by T3, leading to the proposal that T3 causes up-regulation of Wnt-2b in the IMHA, thus playing a crucial role in the regulation of the sensitive period. The action of T3 in the IMM has been ascribed to differential effects on gamma-aminobutyric acid (GABA) receptors, namely sub-types A (ionotropic) and B (metabotropic). From the results of injecting GABA-A and GABA-B agonists and antagonists into the IMM, it was concluded that the T3-dependent sensitive period depends on a balance between the activities of these two receptor subtypes. It is suggested that GABA-B activity is necessary for imprinting and that GABA-A receptor activity suppresses imprinting (Aoki et al., 2018).

### Predispositions

The predisposition of domestic chicks for face-like objects was discovered on account of its resistance to lesions of the IMM, the forebrain region thought to store information about the imprinting stimulus (Horn and McCabe, 1984). It would appear, therefore, that the predisposition is governed by one or more systems outwith the IMM. Mayer et al. (2016) measured c-fos protein expression in chicks that preferred a model of a jungle fowl to a scrambled version of the same model and in chicks having the opposite preference, that is, in chicks that respectively showed a predisposition and those that did not (cf. Johnson and Horn, 1988). No significant difference was found in the hyperpallium apicale (HA, homologous to part of the mammalian visual cortex) or in the tectum [suggested by Johnson (2005) as possibly being one of the regions controlling an analogous predisposition in human neonates]. In the IMM, c-fos protein expression was significantly greater in chicks without the predisposition. The results thus do not implicate the HA or the tectum in the predisposition but indicate that neuronal activity in the IMM is influenced by the predisposition, although it is known that the IMM is not necessary for the predisposition to be expressed. The results raise the interesting possibility that a predisposition is responsible for a net suppression of neuronal activity in the IMM.

A certain amount is known about the properties of the predisposition. It can be induced by mild, non-specific sensory stimulation such as handling, or exposure to white light or a hen’s maternal call (Hampton et al., 1995). There is a sensitive period about 10–40 h after hatching during which the predisposition may be induced (Johnson et al., 1989), and this period can be delayed by general anesthesia (Bolhuis and Horn, 1997) and the noradrenaline-depleting neurotoxin DSP4 (Davies et al., 1992), but DSP4 does not abolish the predisposition (Davies et al., 1985). Preference for the jungle fowl is positively correlated with the concentration of plasma testosterone and can be enhanced further by injection of testosterone (Bolhuis et al., 1986).

The neural basis of the predisposition to follow biological motion has been investigated by c-fos protein immunocytochemistry. Exposure of a chick to a living, behaving conspecific increased expression in the septum and the amygdaloid regions nucleus tenia and arcopallium as compared with chicks that did not experience this exposure (Mayer et al., 2017b). The septum and preoptic area were differentially activated by a living, behaving conspecific in comparison with a rotating model of a conspecific, i.e., not expressing biological motion (Mayer et al., 2017a). Moreover, these two regions were selectively activated by another animacy cue, namely a spontaneous change in speed of an object, compared to constant speed (Lorenzi et al., 2017). Thyroid hormones have also been implicated in this predisposition, by experiments in which T3 was injected into chicks that were imprinted on a rotating object not exhibiting biological motion and then tested by being given a choice between animate and inanimate motion. Exogenous T3, known to extend the sensitive period for imprinting (Yamaguchi et al., 2012), was found also to enhance the preference for biological motion, providing a physiological link between imprinting, the sensitive period for imprinting, and the predisposition to prefer biological motion (Miura et al., 2018).

#### A Predisposition to Learn

A technique for detecting a learning-related change has the potential also to yield evidence for the presence of processes contributing to a predisposition. Many investigations of the role of the IMM in imprinting have enquired whether there is a correlation between a measure of the strength of imprinting – a preference score derived from a choice between the familiar imprinting stimulus and a novel stimulus – and a quantitative measurement of a physiological process. Appropriate choice of training period duration can result in some chicks learning nothing despite exposure to the imprinting stimulus and other chicks becoming strongly imprinted – simply, they learn better. If chicks learning nothing show no significant change in the measurement, if the strongly imprinted chicks show a strong change in the measurement, and there is a significant correlation between the measurement and preference score, one is led to conclude that the measurement is related to learning. One would expect training to have induced a learning-related change if, in addition, residual variance from the correlation (i.e., variance about the regression line) is no lower than the variance of untrained control chicks (McCabe and Horn, 1988; Horn and Johnson, 1989; McCabe, 2013). This is because an effect of training that is related to learning would add to the variance in control chicks and would reveal itself in a significant correlation with preference score; residual variance about the regression line would have the same origin as in untrained chicks. In contrast, a variance about the regression line that is significantly lower than the control variance is evidence, not for an effect of training, but merely a resorting of the control values. For example, chicks with high levels of the physiological measurement could be predisposed to learn well and chicks with low levels of the measurement predisposed to learn poorly. Evidence of this kind for a predisposition was found by Margvelani et al. (2018) when investigating the effect of micro-RNA (miRNA) expression in the IMM. miRNA profiling identified a miRNA (gga-miR-130b-3p) whose expression was negatively correlated with preference score. In addition, the residual variance about the regression line was significantly lower than the variance of untrained control chicks. For the reasons outlined above, it was inferred that this micro-RNA was not affected by training but was present at control levels in poor learners and low levels in good learners. That is, its concentration reflects a predisposition to learn well or badly and is a predictor of how well chicks will learn when trained with an imprinting stimulus – (see Margvelani et al. (2018) for detailed). Interestingly, levels of a protein controlled by this miRNA, cytoplasmic polyadenylation element binding protein 3 (CPEB-3), was positively correlated with preference score (this direction of correlation is expected because miRNA inhibits protein translation), and the data indicate that training affect CPEB-3 level in a learning-related manner. The miRNA, as one of the factors controlling protein level, reflects a predisposition and is not affected by training.

It is not known whether the predisposition to learn referred to above is related to the other predispositions discussed in this review: there are clearly several types of predisposition and their relationships to one another remain to be elucidated. The correlational technique outlined here (see also Margvelani et al., 2018) is a powerful way of determining how inevitable differences between individual animals may predispose the animals to specific types of behavior.

### Transient Preference for Novelty

There has been little investigation of the neural mechanism underlying a temporary preference for slight novelty during the early phase of imprinting. The neural network models of O’Reilly and Johnson (1994) and Bateson and Horn (1994) do not account for this phenomenon, although the latter suggest that this behavior could be simulated by adding habituation to the properties of the input to the recognition layer of the network from the analysis layer. There may be other possibilities, for example, metaplastic modification of the Hebbian synapses in the recognition layer, namely reducing the efficacy of Hebbian modification by recent activation of the synapses involved (Abraham and Richter-Levin, 2018).

### Classification Together of Temporally Juxtaposed Stimuli

The demonstration by lesion studies that the IMM is necessary for both acquisition and retention of a preference acquired through imprinting also revealed a functional difference between the left and right sides of this region. A series of experiments indicated that the left IMM is responsible for long-term storage of a representation of the imprinting stimulus and that the right IMM also has a storage function, but of a different nature. If the left IMM is lesioned shortly after training and the right remains intact for approximately one more day, storage occurs in a region, identified as S′ (Cipolla-Neto et al., 1982), which must lie outside the IMM because the IMM at that point is no longer present. Conversely, if the order of lesioning is reversed, i.e., the right IMM lesioned before the left, the chicks show no memory for the imprinting stimulus: the remaining left IMM is critical for retention of the preference. Thus, S′ becomes functional under the influence of the right (reviewed in Horn, 1985; McCabe, 1991). It is therefore possible to arrange for chicks to be imprinted without S′ becoming operational, by lesioning the right IMM shortly after training. It is also possible for other chicks to possess a functional S′ with no IMM, by lesioning the IMM bilaterally after S′ has become operational. It is then possible to compare the properties of the two memory systems: S′ and the IMM. Presentation of two imprinting stimuli to chicks in close temporal juxtaposition results in behavior indicating that the two stimuli are classified together (Chantrey, 1974, 1976). This may be demonstrated by training chicks with two stimuli presented sequentially according to a random schedule in short intervals of duration 10–30 s and inter-stimulus intervals of 5–25 s (“mixed” training). Chicks trained in this way subsequently learn to discriminate between the two stimuli in a heat-rewarded conditioning procedure, but more slowly than chicks that have been subjected to the two stimuli for the same total time, but in longer, separate intervals, each of duration 53 min (“separate” training) (Honey et al., 1995). These authors found that chicks in which S′ was intact also showed this effect. However, if the chicks became imprinted and S′ was not allowed to become operational, the inferred ability to classify stimuli together was lost (Honey et al., 1995). It was concluded that the IMM system can store information about the imprinting stimulus, but S′ is required for the flexibility of processing that permits classification together.

#### Recognition of Individuals

Johnson and Horn (1987) found that chicks can learn to distinguish between two different jungle fowl models after being imprinted to one of them; moreover, this ability was abolished by lesions to the IMM. Town (2011a) socially reared chicks in groups of six and then recorded the responses of IMM neurons, in these chicks, to video recordings of familiar and unfamiliar chicks in groups, in the presence of conspecific calls. Note that both testing and social rearing involved simultaneous visual and auditory stimulation. Under these conditions, neuronal responsiveness to the familiar chicks was lower than to novel chicks, this effect predominating in the right IMM. Although a group of chicks rather than an individual animal was used in this experiment, the results provide evidence of remarkable learning-dependent discrimination between naturalistic stimuli, such as may be engaged in learning the features of an individual. As noted previously, responsiveness of IMM neurons to the visual and auditory components of a familiar audio-visual imprinting stimulus are different (Nicol and Horn, 2009). Responsiveness to a bisensory stimulus may be different again and not necessarily in linear combination of the constituent modalities.

The question of the IMM’s responsiveness to stimuli sharing only some of the features of an imprinting training stimulus was addressed by Town and McCabe (2011). In these experiments, chicks were trained with an artificial visual stimulus accompanied by a maternal call, followed by determination of IMM neurons’ responsiveness to combinations of familiar and novel versions of the visual and auditory components of the training stimulus. As reported previously (e.g., Brown and Horn, 1994), neuronal responsiveness to the visual component of the training stimulus was increased by imprinting, whereas responsiveness to a novel visual stimulus was not. Responsiveness to unisensory auditory stimuli was equivocal: there was a significant interaction between stimulus familiarity and training condition but no clear indication of how either of these factors contributed, possibly because of the small number of animals involve. A particularly strong increase in responsiveness was observed when the familiar visual stimulus was presented with a novel maternal call, leading to the suggestion that IMM neurons may be sensitive to changes in the context of a familiar visual stimulus (Town and McCabe, 2011). It is also apparent from these results that a response to a bisensory stimulus is not necessarily the sum of responses to its unisensory components: there can be considerable interaction between the underlying processes.

Despite the obvious need for caution in comparing neuronal activity in the IMM with behavior arising from imprinting and despite the different timescales involved, there is a noteworthy parallel between increased neuronal responsiveness to a familiar visual stimulus in a novel auditory context (Town and McCabe, 2011) and the behavioral preference for slight novelty observed in the early stages of imprinting (Jackson and Bateson, 1974; Bateson and Jaeckel, 1976). Such behavior was incorporated into the model of Bateson (1973, 1974). Bateson and Horn (1994) consider such behavior when discussing their neural network model, postulating, in addition to the formal implementation of the model, attenuation of input into recognition modules as a result of continuous exposure to the same stimulus. Bateson (2000) proposed a similar addition to the model in the light of experiments investigating chicks’ classification together of imprinting stimulus features (Honey and Bateson, 1996).

## Imprinting to Several Objects and Relational Concept Learning

Neurobiological analysis has yet to make headway with these behavioral phenomena, important though they undoubtedly are in the life of a young animal and at least implied by existing neural network models (Martinho and Kacelnik, 2016).

## Conclusion

By establishing a social bond between a newly hatched chick and a potentially protective conspecific adult, imprinting can substantially increase the chick’s chances of survival. The contribution of imprinting to social cohesion is therefore of great biological importance. Imprinting is also experimentally tractable. Therefore, much is known about its behavioral characteristics and the underlying neural mechanisms. Modeling the behavior associated with imprinting has yielded useful insights and predictions at the behavioral level, but such models also require physiological validation, which currently is incomplete. If such validation can be accomplished, the relevant models may make an important contribution to understand social behavior at the physiological level.

## Author Contributions

The author confirms being the sole contributor of this work and has approved it for publication.

### Conflict of Interest Statement

The author declares that the research was conducted in the absence of any commercial or financial relationships that could be construed as a potential conflict of interest.
